# A novel nomogram model of breast cancer-based imaging for predicting the status of axillary lymph nodes after neoadjuvant therapy

**DOI:** 10.1038/s41598-023-29967-1

**Published:** 2023-04-12

**Authors:** Pengyu Zhang, Xiang Song, Luhao Sun, Chao Li, Xiaoyu Liu, Jiaying Bao, Zhaokun Tian, Xinzhao Wang, Zhiyong Yu

**Affiliations:** 1 Shandong First Medical University and Shandong Academy of Medical Sciences, Jinan, China; 2 Breast Cancer Center, Shandong Cancer Hospital and Institute, Shandong First Medical University and Shandong Academy of Medical Sciences, Jinan, China; 3 REMEGEN, LTD, 58 Middle Beijing Road, Yantai Economic & Technological Development Area, Yantai, Shandong China; 4 First Clinical Medical College, Shandong University of Traditional Chinese Medicine, Jinan, China

**Keywords:** Cancer, Oncology

## Abstract

This study is aimed to develop and validate a novel nomogram model that can preoperatively predict axillary lymph node pathological complete response (pCR) after NAT and avoid unnecessary axillary lymph node dissection (ALND) for breast cancer patients. A total of 410 patients who underwent NAT and were pathologically confirmed to be axillary lymph node positive after breast cancer surgery were included. They were divided into two groups: patients with axillary lymph node pCR and patients with residual node lesions after NAT. Then the nomogram prediction model was constructed by univariate and multivariate logistic regression. The result of multivariate logistic regression analysis showed that molecular subtypes, molybdenum target (MG) breast, computerized tomography (CT) breast, ultrasound (US) axilla, magnetic resonance imaging (MRI) axilla, and CT axilla (all *p* < 0.001) had a significant impact on the evaluation of axillary lymph node status after NAT. The nomogram score appeared that AUC was 0.832 (95% CI 0.786–0.878) in the training cohort and 0.947 (95% CI 0.906–0.988) in the validation cohort, respectively. The decision curve represented that the nomogram has a positive predictive ability, indicating its potential as a practical clinical tool.

## Introduction

Breast cancer is the most common malignant tumor in women worldwide and it is the leading cause of death from cancer. It has attracted worldwide attention because of its increasing incidence, especially in the younger population^1^. Neoadjuvant therapy (NAT) is the major treatment for patients with advanced localized breast cancer and for patients whose tumor size is too large to opt for breast conservation at an early stage^2,3^. With an increasing number of publications on NAT and clinical trials, the number of breast cancer patients undergoing preoperative therapy is growing^4–6^, resulting in degradation in the tumor stage and significant improvement in the rate of breast conservation. In addition, NAT has been shown to improve the axillary lymph node stage in patients with axillary lymph node metastases from breast cancer, leading to pathologic complete response (pCR) in 50–60% of patients and thereby prolonging the survival time^7,8^. Axillary lymph node dissection (ALND) may not be necessary for patients who obtain axillary pCR after NAT. As an alternative to surgery, sentinel lymph node biopsy (SLNB) has been introduced as a standard diagnostic procedure for breast cancer since 2005, which accurately predicts axillary lymph node status^9^. The American College of Surgeons Oncology Group 1071 and the SENTINA trials found that ALND can be avoided when fewer than three sentinel lymph nodes are positive, which can prevent complications such as lymphedema and upper limb numbness^7,10^. However, the pathology results can only be obtained after surgical resection rendering the early evaluation of the efficacy of chemotherapy difficult. Moreover, the overall false-negative rate of these studies was 5.4–14%^11^. Therefore, to avoid ALND after NAT, it is crucial to accurately evaluate the axillary lymph node status. Evaluation of lymph node status after NAT is also key to providing essential information thereby guiding treatment and prognosis of breast cancer.

Currently, non-invasive imaging methods for evaluating axillary lymph node status after NAT mainly include mammography, ultrasound (US), magnetic resonance imaging (MRI), and positron emission tomography/computed tomography (PET/CT). With the current techniques, imaging detection of the primary lesions of breast cancer is very mature, but whether it can reflect the pathological condition of the axilla remains controversial. In recent years, radiomics and deep learning have shown promising performance in evaluating the status of axillary lymph nodes after NAT^12–14^. However, due to the influence of image acquisition methods, image quality, and lacking unified research standards, they cannot be widely used in clinical practice. Furthermore, few studies proposed to predict the axillary lymph nodes after NAT by constructing a nomogram. Nomogram integrates multiple factors and transforms the complex regression equation into a simple visual figure, which is more convenient for accurately evaluating the patient's condition. However, there are limitations in the existing nomogram studies, such as the incomplete data and predictive factors hence necessitating further research to improve it. Moreover, there is an urgent need to develop novel non-invasive tools with improved accuracy for predicting axillary pCR after NAT, thereby reducing the incidence of postoperative complications.

Briefly, the purpose of this study is to establish a new nomogram based on imaging and clinical indicators to accurately predicate the status of axillary lymph nodes after NAT and to provide some references for clinicians to make objective and effective treatment plans.

## Results

### Baseline characteristics of the study population

This study retrospectively enrolled a total of 410 patients who experienced NAT and surgical treatment for breast cancer. Among them, 186 patients (45.9%) showed axillary pCR in the pathological examination, and 224 patients (54.6%) presented residual axillary lymph node lesions after breast surgery. The whole cohort was randomly divided into the training cohort of 302 patients and the validation cohort of 108 patients at a 3:1 ratio to construct and validate the nomogram model. All the baseline variables in the two groups are summarized in Table 1.

**Table 1:** Baseline features between patients with or without axillary pCR.(At 3.1).

	Training cohort N = 302 (%)			Validation cohort N = 108 (%)		
	Axillary pCR n = 133	Axillary non pCR n = 169	*p* value	Axillary pCR n = 53	Axillary non pCR n = 55	*p* value
Age (years)			0.839			0.863
> 49	74 (24.5)	96 (31.8)		29 (26.9)	31 (28.7)	
≤ 49	59 (19.5)	73 (24.2)		24 (22.2)	24 (22.2)	
Trastuzumab treatment			0.069			0.179
Yes	56 (18.5)	54 (17.9)		23 (21.3)	17 (15.7)	
No	77 (25.5)	115 (38.1)		30 (27.8)	38 (35.2)	
Chemo regime			0.388			0.687
FEC	13 (4.3)	15 (5.0)		4 (3.7)	5 (4.6)	
Taxane	14 (4.6)	27 (8.9)		2 (1.9)	4 (3.7)	
Both	106 (35.1)	127 (42.1)		47 (43.5)	46 (42.6)	
Pre-treatment clinical T stage			0.695			0.757
T1	23 (7.6)	37 (12.3)		13 (12.0)	13(12.0)	
T2	88 (29.1)	107 (35.4)		30 (27.8)	27 (25.0)	
T3	16 (5.3)	16 (5.3)		9 (8.3)	13 (12.0)	
T4	6 (2.0)	9 (3.0)		1 (1.0)	2 (1.9)	
Molecular subtypes			< 0.001			< 0.001
Luminal A	10 (3.3)	41 (13.6)		3 (2.8)	15 (13.9)	
Luminal B	65 (21.5)	97 (32.1)		20 (18.5)	33 (30.6)	
HER2 positive	43 (14.2)	23 (7.6)		19 (17.6)	5 (4.6)	
TNBC	15 (5.0)	8 (2.6)		11 (10.2)	2 (1.9)	
Pathological response of breast tumor			< 0.001			0.005
Complete response	63 (20.9)	19 (6.3)		21 (19.4)	7 (6.5)	
Near complete	31 (10.3)	52 (17.2)		12 (11.1)	26 (24.1)	
Partial response	20 (6.6)	47 (15.6)		13 (12.0)	12 (11.1)	
Minimal response	17 (5.6)	35 (11.6)		7 (6.5)	10 (9.3)	
No response	2 (0.7)	16 (5.3)		0 (0)	0 (0)	
MG breast			< 0.001			< 0.001
Complete response	93 (30.8)	81 (26.8)		44 (40.7)	27 (25.0)	
Not complete response	40 (13.2)	88 (29.1)		9 (8.3)	28 (25.9)	
CT breast			< 0.001			< 0.001
Complete response	87 (28.8)	57 (18.9)		34 (31.5)	8 (7.4)	
Not complete response	46 (15.2)	112 (37.1)		19 (17.6)	47 (43.5)	
US axilla			< 0.001			< 0.001
Normal	73 (24.2)	42 (13.9)		32 (29.6)	13 (12.0)	
Abnormal	60 (19.9)	127 (42.1)		21 (19.4)	42 (38.9)	
MRI axilla			< 0.001			< 0.001
Normal	83 (27.5)	61 (20.2)		38 (35.2)	9 (8.3)	
Abnormal	50 (16.6)	108 (35.8)		15 (13.9)	46 (42.6)	
CT axilla			< 0.001			< 0.001
Normal	65 (21.5)	33 (10.9)		27 (25.0)	5 (4.6)	
Abnormal	68 (22.5)	136 (45.0)		26 (24.1)	50 (46.3)	

*FEC* fluorouracil + epirubicin + cyclophosphamide.

### Selection of factors for constructing the model

After univariate analysis, the variables involved in the multivariate logistic regression analysis were molecular subtype, breast US, molybdenum target (MG) breast, CT breast, US axilla, MG axilla, MRI axilla, and CT axilla. The multivariate analyses demonstrated that the possibility of axillary pCR after NAT was significantly correlated with molecular subtypes (p < 0.001), MG breast (p < 0.001), CT breast (p < 0.001), US axilla (p < 0.001), MRI axilla (p < 0.001), CT axilla (p < 0.001). However, the US breast and MG axilla were not statistically significant, as shown in Table 2.

**Table 2:** Multivariate logistic regression analysis.

Characteristics	Training cohort		Validation cohort	
	OR (95% CI)	*p* value	OR (95% CI)	*p* value
Molecular subtypes				
TNBC	Ref		Ref	
Luminal A	0.130 (0.043–0.392)	< 0.001	0.139 (0.038–0.510)	0.003
Luminal B	0.130 (0.055–0.307)	< 0.001	0.134 (0.050–0.356)	< 0.001
HER2 positive	0.364 (0.170–0.778)	0.009	0.383 (0.163–0.902)	0.028
MG breast				
Abnormal	Ref		Ref	
Normal	0.396 (0.245–0.638)	< 0.001	0.414 (0.235–0.731)	0.002
CT breast				
Abnormal	Ref		Ref	
Normal	0.269 (0.167–0.434)	< 0.001	0.423 (0.231–0.773)	0.005
US axilla				
Abnormal	Ref		Ref	
Normal	0.272 (0.167–0.443)	< 0.001	0.378 (0.213–0.671)	0.001
MRI axilla		< 0.001		
Abnormal	Ref		Ref	
Normal	0.254 (0.152–0.423)	< 0.001	0.387 (0.201–0.743)	0.004
CT axilla				
Abnormal	Ref		Ref	
Normal	0.340 (0.212–0.545)	< 0.001	0.343 (0.194–0.604)	< 0.001

### Predictive nomogram for the probability of axillary pCR after NAT

**Figure 1:**
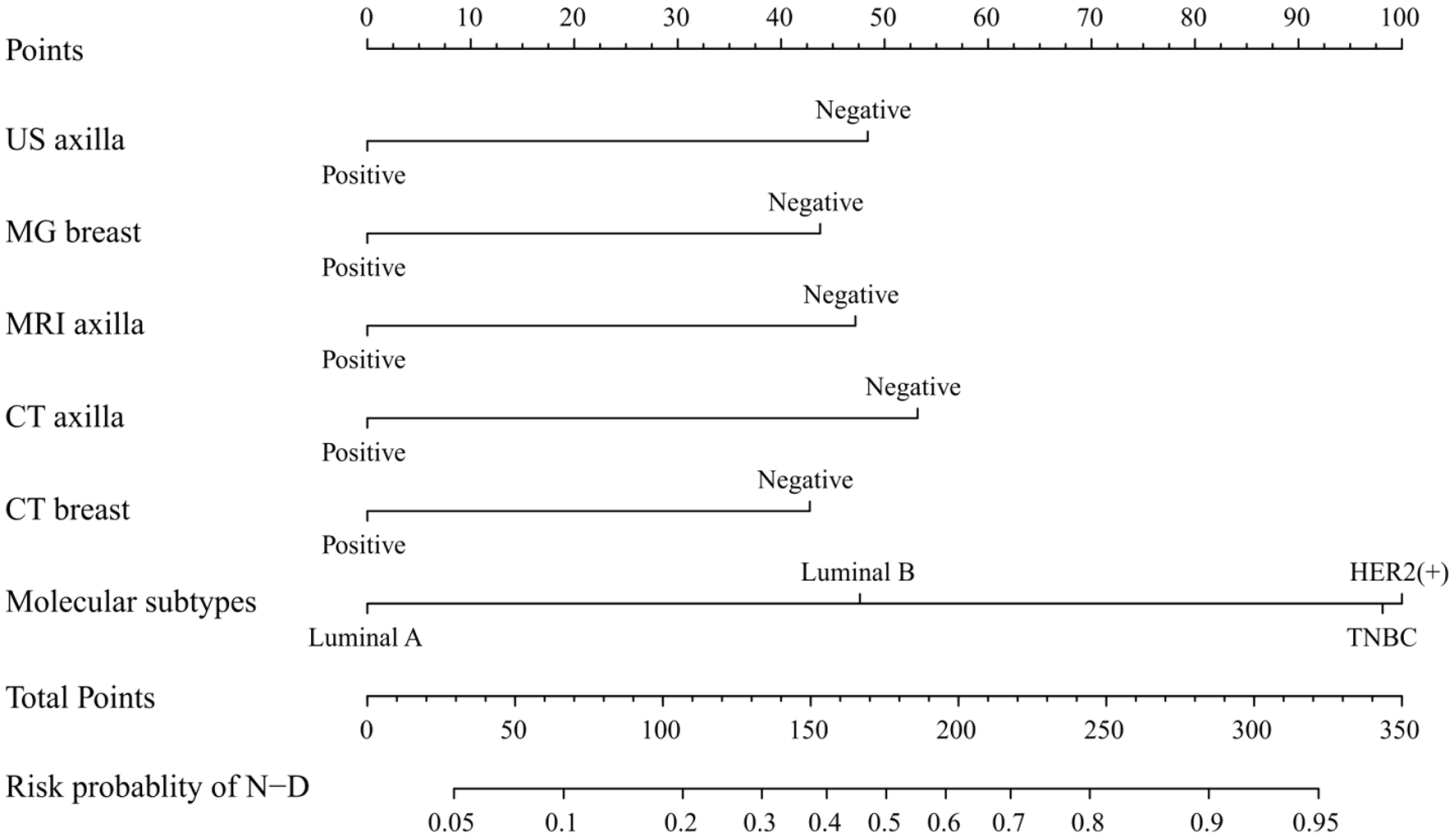
Nomogram to predict the individual probability of axillary nodal pCR after NAT. *Assessed on core biopsy before neoadjuvant therapy.

Based on the final regression analysis, we included six important risk factors that showed significant association with axillary pCR after NAT in the multivariate regression analysis and constructed a nomogram for predicting the same (Fig. 1). The total scores were calculated based on molecular subtypes, MG breast, CT breast, US axilla post-chemo, MRI axilla post-chemo, and CT axilla post-chemo. The values of these variables correspond to a fraction on the fractional scale axis and the total score is calculated by the addition of individual scores. Through the projection of the total score to a lower total scale score, it is possible to predict pCR in the posterior axilla of NAT.

### Nomogram validation

**Figure 2:**
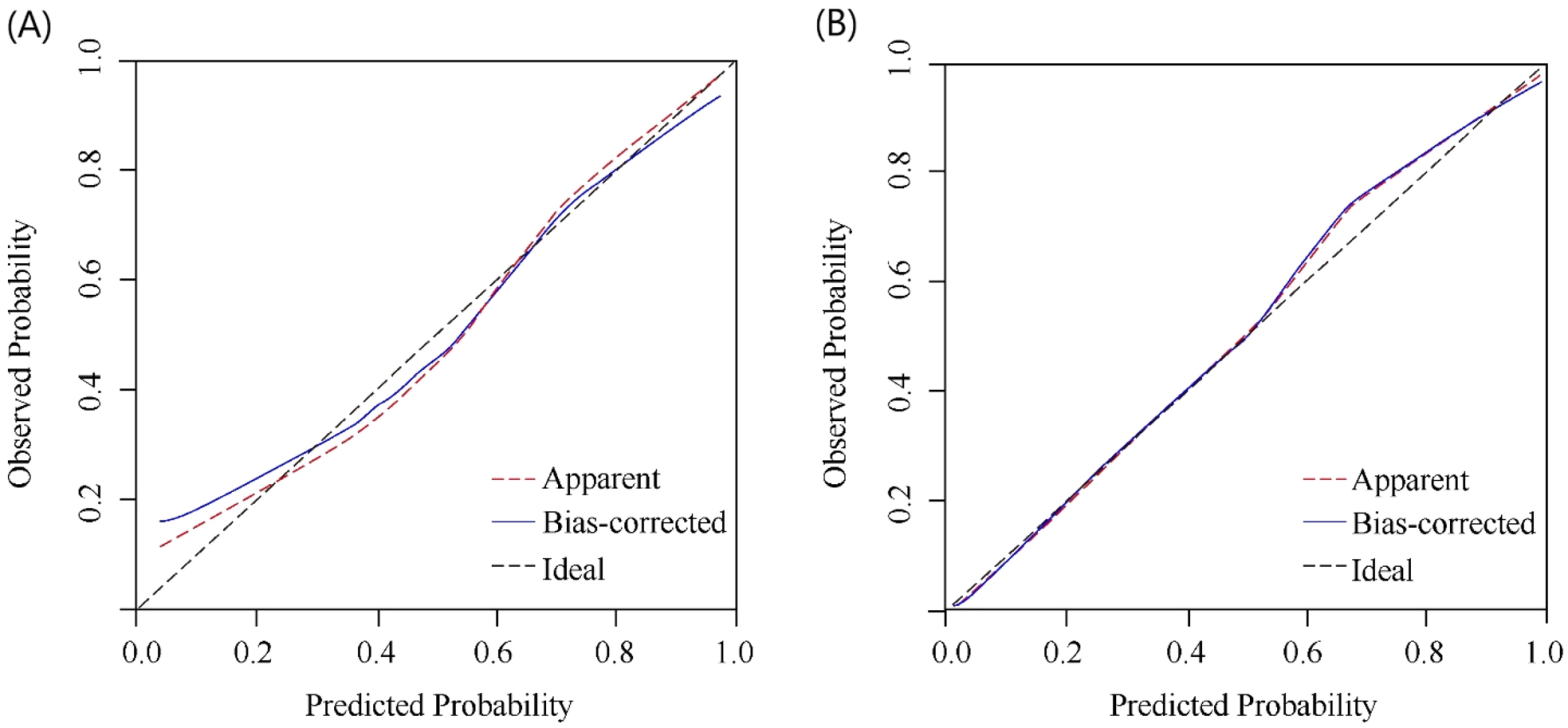
Calibration curves of the nomogram in the training (**A**) and validation (**B**) cohort.

**Figure 3:**
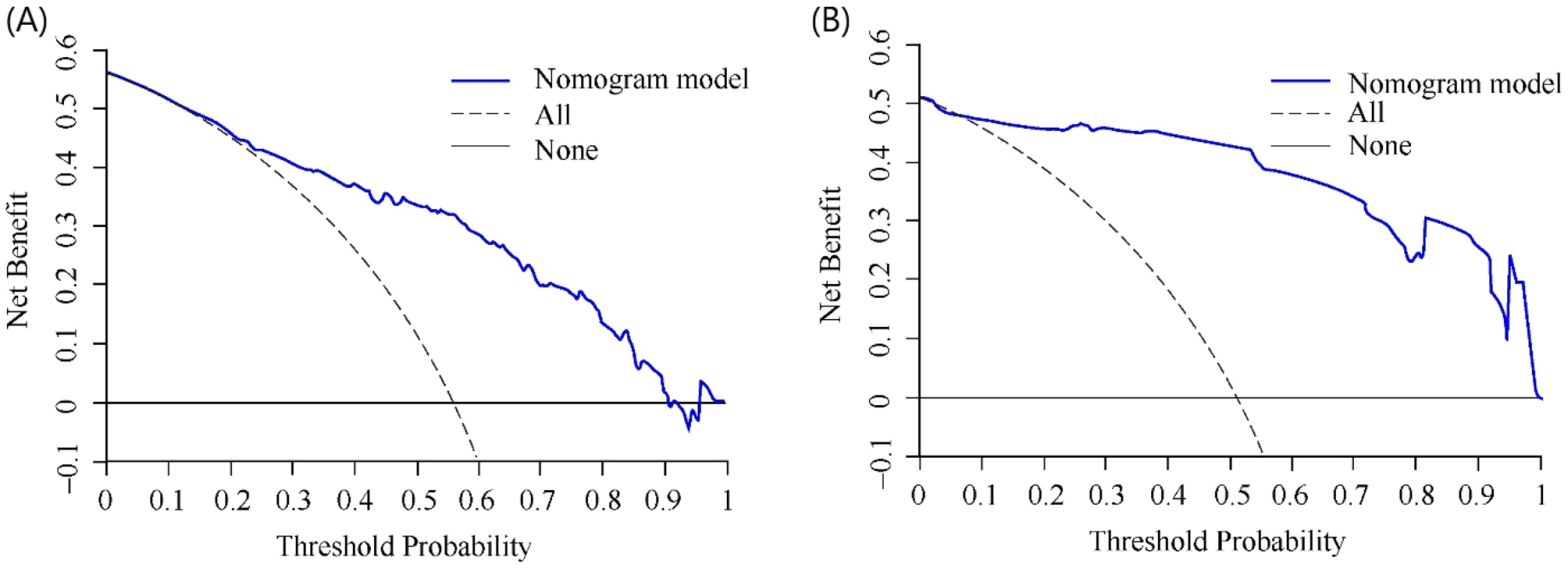
Figure shows the results of the decision curve analysis for the nomogram model. (**A**) The decision curve of the training cohort, and (**B**) is the verification cohort. The clinical utility of the nomogram prediction model was greater when the threshold probability was between 0.44 and 0.68 or greater than 0.78.

In the training cohort, the C index of the axillary pCR response was 0.832 (95% CI 0.786–0.878), indicating that the nomogram showed good discriminative power. Next, we evaluated the model performance by establishing the internal and external calibration curves based on the training and validation cohorts, respectively (Fig. 2). The results showed that the prediction performance of the constructed nomogram model correlated well with the actual status of axillary pCR. These results further confirmed the reliability of the nomogram model established based on the combination of clinical factors and imaging indicators. In addition, according to the decision curve analysis results (Fig. 3), when the threshold probability of patients was between 0.44 and 0.68, or greater than 0.78, the use of nomogram can obtain greater benefit, which has beneficial utility in clinical application.

### ROC analysis for the comparison of the novel nomogram model with various imaging diagnostics and molecular subtypes

**Figure 4:**
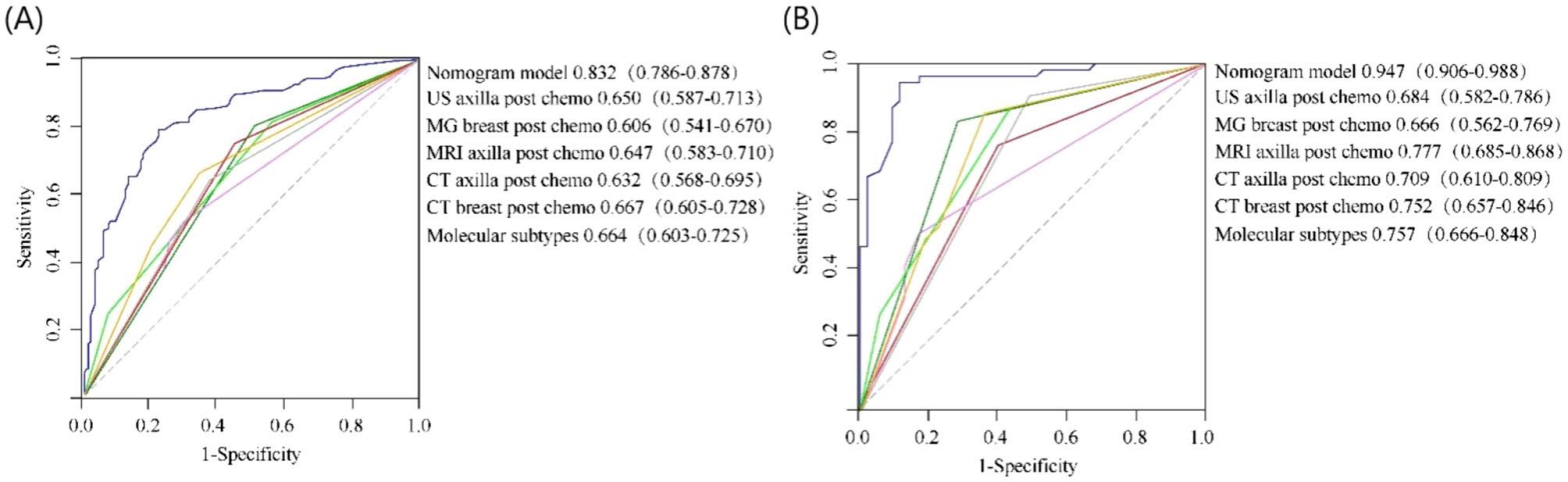
The ROC curve of the nomogram, with an AUC of 0.832 in the training cohort (**A**) and an AUC of 0.947 in the validation cohort (**B**).

Next, we compared the predictive performance of the constructed nomogram with current-generation imaging diagnostics and molecular subtypes using ROC analysis. The ROC curve for predicting axillary pCR after NAT in breast cancer patients in the training cohort is shown in Fig. 4. In the ROC curve of the training cohort, the AUC value of the nomogram for predicting the pCR of axillary lymph nodes after NAT was 0.832 (95% CI 0.786–0.878). The AUC values of US axilla, MG breast, MRI axilla, CT axilla, CT breast and Molecular subtypes are 0.650 (95% CI 0.587–0.731), 0.606 (95% CI 0.541–0.670), 0.647 (95% CI 0.583–0.710), 0.632 (95% CI 0.568–0.695), 0.667 (95% CI 0.605–0.728), and 0.664 (95% CI 0.603–0.725), respectively. In the ROC curve of the verification cohort, the AUC value of the nomogram for predicting the pCR of axillary lymph nodes after NAT was 0.947 (95% CI 0.906–0.988). The AUC values of US axilla, MG breast, MRI axilla, CT axilla, CT breast and Molecular subtypes are 0.684 (95% CI 0.582–0.786), 0.666 (95% CI 0.562–0.769), 0.777 (95% CI 0.685–0.868), 0.709 (95% CI 0.610–0.809), 0.752 (95% CI 0.657–0.846), and 0.757 (95% CI 0.666–0.848), respectively. In both the training and validation cohorts, the constructed nomogram model showed significantly higher AUC values compared with various imaging diagnostic indicators and molecular subtypes. These results clearly show that the nomogram developed in this study has better prediction performance and discrimination for axillary pCR after NAT.

## Discussion

In this study, we constructed a nomogram based on various imaging diagnostics and clinical indicators to predict the status of axillary lymph nodes after NAT. We identified 6 pretreatment factors as independent predictors of axillary pCR namely molecular subtypes, MG breast, CT breast, US axilla, MRI axilla, and CT axilla. Analyses in the training and the validation cohorts showed that the nomogram could predict the axillary pCR after NAT. In addition, the calibration curve demonstrated that the predictive model had satisfactory calibration. Collectively, our results show that the novel constructed nomogram is a reliable and relatively objective nomogram with good clinical utility that could facilitate the pretreatment prediction of axillary pCR after NAT.

In this study, the molecular subtypes of breast cancer are proved to be an independent predictor (p < 0.001) of axillary lymph node status. The molecular subtype is an important index to predict the primary treatment focus for breast cancer patients after NAT. Further, molecular subtypes of axillary lymph nodes are also considered to be an important index and hence it is meaningful to be enrolled as a predictive factor in the nomogram model. In previous studies, several nomograms have been developed to predict axillary lymph node status in breast cancer patients undergoing NAT^15–18^. These studies included clinical indicators such as ER, PR, HER2, and Ki67 which showed good performance as independent predictors. Of note, these conclusions are also consistent with the results of the present study. Our results indicate that the molecular subtypes play a crucial role in the prediction of axillary lymph node involvement after NAT and are worthy of attention by clinicians.

Our study predicted the status of axillary lymph nodes through the diagnosis and changes of imaging examination before and after NAT. In our study, the US axilla, MRI axilla, and CT axilla are independent predictors (p < 0.001), and the MG axilla is not an independent predictor (p > 0.05). In previous studies on US, MG, MRI, and CT; US and MRI are found to be the best in predicting the status of axillary lymph nodes unlike MG, which could not accurately predict it^19,20^. These findings are also consistent with the results of this study. Moreover, there are also studies on the establishment of nomogram prediction models through in-depth learning of US or MRI, or simply through clinical indicators. The AUC of the training cohort and validation cohort in the previous US-based deep learning nomogram model is 0.816 and 0.759 respectively^21^. Further, the MRI-based nomogram model had an AUC of 0.81^22,23^, and the AUC of the clinical indicators-based nomogram model was 0.802^24^. In our study, we used four kinds of imaging methods to establish the nomogram prediction model in combination with clinical factors. The AUC of the training cohort and the validation cohort is 0.832 and 0.947, respectively, higher compared to the above-mentioned studies. This indicates that the constructed nomogram model had good diagnostic performance and is better than previous nomogram studies which relied on single imaging indicators or only clinical indicators.

In addition, we also found that the change in the primary lesion of breast cancer could also predict the status of the axillary lymph node. We found that both MG breast post-chemo and CT breast post-chemo were independent predictors of axillary lymph node status after NAT. Previous studies have focused on the size of the primary lesion as a factor to predict the axillary lymph node status^25–27^. However, breast cancer usually shows a decrease in the number of tumor cells after NAT, which is not always reflected by volume. In this study, we included the changes in primary breast cancer before and after NAT and divided them into complete response and not complete response groups according to the RECIST criterion. We found that if the primary lesion reached CR, the probability of axillary lymph nodes reaching pCR was 60.4%, and if the primary lesion was not reaching CR, the probability of axillary lymph nodes not reaching pCR was 70.9%. This suggests that it is feasible to predict the status of axillary lymph nodes by assessing whether the primary lesion reaches CR or not. Interestingly, in our study, the primary changes reflected by US and MRI had no statistical significance for the prediction of axillary lymph node status (p > 0.05). We speculate that the sample size of this study is not large enough and further research is warranted to comprehensively understand this in the future.

Although the nomogram developed in this study showed better prediction performance than the existing prediction systems, some limitations remain. First, our study is limited to a single-center retrospective study, and it is essential to expand these investigations and use data from other centers for further external verification. Secondly, the sample size included in this study is relatively small, and hence it is necessary to further verify the model’s prediction performance in a larger sample size. Finally, because the main parameters of the model are clinical indicators and imaging results, it may be necessary to apply some specific techniques (such as immunodiagnostic biomarkers) to improve the accuracy of our nomogram. Future research addressing the above-said limitations is essential to validate our nomogram model for wider Utility.

## Conclusions

This new nomogram prediction model based on clinical indicators and imaging can predict the status of axillary lymph nodes in breast cancer patients after NAT. It is expected to be a practical, non-invasive tool for clinical application. Patients with negative SLND after NACT may also have axillary lymph node metastases^11^, and this model can help detect whether these patients have axillary lymph node metastases after NACT. If no metastasis is found by both SLND and nomogram, axillary dissection may be avoided. However, the sample size still needs to be expanded in the future.

## Materials and methods

### Study population

**Figure 5:**
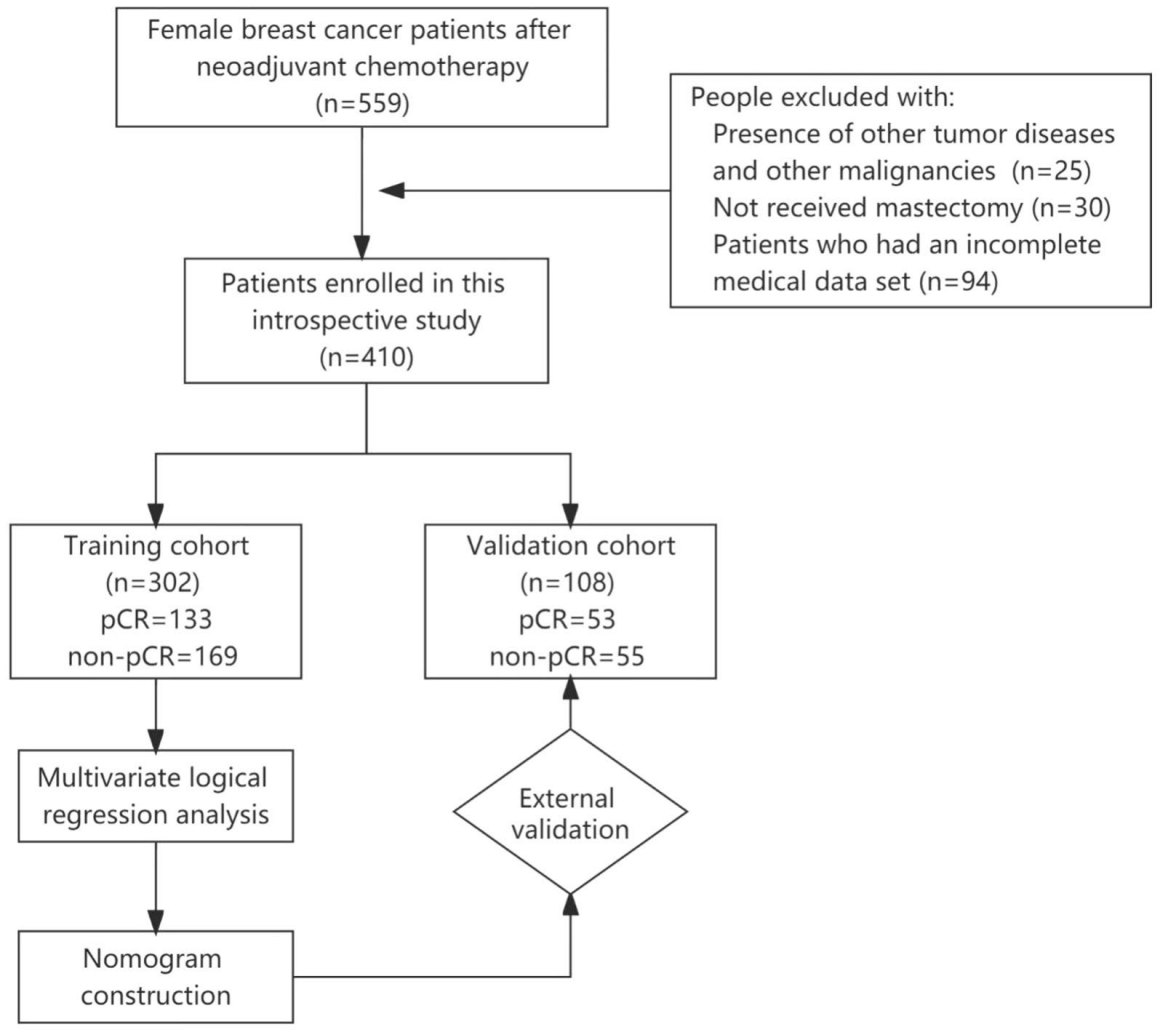
Flowchart of the current study design.

This retrospective study was approved by the ethics committee of Shandong Frist Medical University and Shandong Academy of Medical Sciences (SDTHEC2022003131). All of our studies were conducted with the informed consent of patients, and all patient data were anonymised. Our work is in accordance with the STROBE Observational Research Guidelines and the Helsinki Declaration. The clinical and pathological data of 559 breast cancer patients were retrospectively collected from Shandong Cancer Hospital and Institute in China from January 2015 to December 2020. We screened the data using the following inclusion criteria: (1) older than 18 years; (2) axillary positive before NAT; (3) NAT was performed for 6 to 8 cycles before operation; (4) underwent surgical resection with pathological confirmation, SLNB and ALND were performed; (5) immunohistochemical indicators including ER, PR, HER2, and Ki-67 available. Breast cancer was staged based on the American Joint Committee on Cancer (AJCC, 7th edition, 2010) and subdivided into four subtypes according to St Gallen International Expert Consensus on the Primary Therapy of Early Breast Cancer 2013^28^. Exclusion criteria were: (1) the presence of other tumors and malignancies; (2) patients who have not received a mastectomy; and (3) patients who had incomplete medical data. Finally, 410 patients were included in the study. The eligible people were randomly divided into training and validation cohorts at a 3:1 ratio. The research flow chart is shown in Fig. 5. The study was approved by the Ethics Committee of Shandong Cancer Hospital and Institute (registration number: SDTHEC2022003131).

### Evaluating the safety and efficacy of NAT

According to Response Evaluation Criteria In Solid Tumors (RECIST) criteria (version 1.1), the response to NAT is divided into four levels: complete response (CR), partial response (PR), progressive disease (PD), stable disease (SD)^29^. According to the changes in the primary breast cancer before and after NAT, the patients were divided into two groups: the complete response group and the not complete response group. The not complete response group was PR, SD, and PD. In addition, we divided the pathological response of primary tumors after NAT into complete response, near complete, partial response, minimal response, and no response according to the chemotherapy response score (CRS)^30^.

### Endpoints and study design

The primary purpose of this study is to determine the independent predictors of pCR in the posterior axilla after NAT and to develop and externally validate a nomogram model based on the predictors. The whole study population was divided into two different cohorts: a training cohort of 302 patients for nomogram development and a verification cohort of the remaining 108 patients for external verification of nomogram. As endpoints of the study, all patients were divided into the following two groups: patients with axillary lymph nodes reaching pCR and patients with axillary residual lymph nodes after NAT. Finally, a prediction model in the form of a nomogram was developed to predict the axillary state after NAT.

### Statistical analysis

The chi-squared test (χ^2^) was used to compare the classified variables. The risk factors of pathological reactions were analyzed by univariate and multivariate logistic analysis. The nomogram was drawn according to the results of multivariate logistic regression analysis. Based on multivariate logistic regression, the ratio of each regression coefficient ranging from 0 to 100 points is converted into a nomogram. The influence of the variable with the highest β coefficient (absolute value) is assigned 100 points. Then we added the points of all independent variables to produce a total, which is then converted to the predicted probability. The diagnostic performance of each model was evaluated by the receiver operating characteristic (ROC) curve analysis, and the area under the ROC curve (AUC) was calculated as a comparative index. In general, C-index and AUC values above 0.6 indicate a reasonable estimate. Statistical analysis and mapping were carried out with the software IBM SPSS20.0 (SPSS Inc, Armonk, NY) and R 3.1.2 (The R Foundation for Statistical Computing, Vienna, Austria) with rms statistical package 20. p < 0.05 is considered statistically significant.

### Informed consent

Informed consent was obtained from all subjects/legal guardians.

### Ethics approval and consent to participate

This retrospective study was approved by the ethics committee of Shandong Frist Medical University and Shandong Academy of Medical Sciences (SDTHEC2022003131).

## Data Availability

The raw data of this paper are available upon reasonable request to the corresponding author.

## Abbreviations

NAT: Neoadjuvant therapy
pCR: Pathological complete response
MG: Molybdenum target
PET/CT: Positron emission tomography/computed tomography
US: Ultrasound
MRI: Magnetic resonance imaging
ALND: Axillary lymph node dissection
SLNB: Sentinel lymph node biopsy
CR: Complete response
PR: Partial response
PD: Progressive disease
SD: Stable disease
CRS: Chemotherapy response score
ROC: Receiver operating characteristic curve
AUC: Area under the ROC curve
